# Tinea Blepharociliaris: A Case Report

**DOI:** 10.7759/cureus.94274

**Published:** 2025-10-10

**Authors:** Siyu Chen, Jierui Wang, Li Changqiang, Yao Zhang

**Affiliations:** 1 Dermatology and Venereology, Affiliated Hospital of Southwest Medical University, luzhou, CHN

**Keywords:** blepharitis, dermatophyte eyelid infection, dermatophytes, microbiological examination, periocular fungal infection

## Abstract

Blepharitis is a prevalent inflammatory disease of the ocular surface, traditionally associated with bacterial infections or helminth mite parasites. However, recent studies have demonstrated that dermatophyte infections of the eyelids can trigger fungal blepharitis, and this condition can be misdiagnosed as eczema or bacterial infections due to its atypical clinical manifestations. In this article, we present a case of fungal blepharitis in a 25-year-old female patient, an isolated brown crust around the eye (atypical lesion), and a six-month history of possible exposure to contaminated cosmetics were the key features distinguishing the case from a bacterial infection. The patient had been administered glucocorticoids inappropriately, resulting in the migration of the lesion. The diagnosis was confirmed through microscopic examination, which revealed the presence of rod-shaped, large, and small spores. The condition was resolved through the administration of oral terbinafine for two weeks. This case emphasizes that tracing the source of contact and identifying subtle signs (e.g., sesame scab) in the medical history are key to the early diagnosis of fungal eyelid infection and to avoid the chronicity caused by misdiagnosis. When determining the cause of blepharitis, particular attention should be paid to a history of recalcitrance to conventional therapy and the presence of atypical lesions, such as brownish crusts.

## Introduction

Blepharitis is defined as an inflammatory disease that occurs at the edge of the eyelid and its adjacent areas (especially near the root of eyelashes). It has been observed to persistently affect patients' health for extended periods, manifesting in complex eyelid symptoms. The common symptoms associated with blepharitis include itching, crusting, burning sensation, irritation, lacrimation, photophobia, blurred vision, and redness of the eyes [1]. The symptoms associated with blepharitis manifest in a variety of ways, depending on the specific area affected. Anterior blepharitis, for instance, affects the outer edge of the eyelid, while posterior blepharitis affects the inner edge. Blepharitis is mainly related to bacterial infection. A study revealed that the most prevalent bacteria observed in patients diagnosed with blepharitis are *Staphylococcus epidermidis*, *Staphylococcus aureus*, and *Corynebacterium *spp. [2]. Anterior blepharitis is generally *Staphylococcus* or seborrheic, while posterior blepharitis is caused by meibomian gland dysfunction [3]. A chronic inflammatory environment caused by blepharitis may create susceptible conditions for chronic infections. In addition to bacterial infection, some fungi (e.g., yeast, mold, and dermatophyte) can also cause blepharitis. For example, facial dermatophyte infection can affect the eyelid and cause fungal blepharitis, tinea faciei, tinea blepharociliaris, and periocular tinea are ringworm infections of the face, eyelids, and eyelashes and eyelids alone, respectively [4]. If left untreated, blepharitis may lead to more serious ocular problems. For instance, the inflammation may disseminate to the conjunctiva, precipitating conjunctivitis, and may affect the cornea, resulting in keratitis, which in turn affects vision with serious consequences [5], underscoring the importance of early diagnosis and treatment of blepharitis.

The dermatophytes are keratinophilic fungi that cause dermatophytosis (or tinea) by their ability to degrade keratin in the skin, nail, and hair [6]. Dermatophytosis is a prevalent superficial fungal infection that is most often caused by *Trichophyton rubrum*. The areas most affected are the feet, trunk, and nails [7]. The following characteristics are typically observed in such lesions: central clearing, surrounded by an advancing red, scaly, raised border. Vesicles can also be present on the borders of affected areas, allowing for increased inflammation. Local factors, such as long-term use of cosmetics, immunosuppressive status, contact history with diseased animals, excessive moisture or closed clothing, and exposure to pathogenic fungi, may constitute risk factors that should be paid special attention to in the medical history.

Tinea blepharociliarisis a rare fungal blepharitis caused by fungal infection of the eyelid margin and eyelashes. Its clinical manifestations often manifest as non-specific blepharitis, such as itching, erythema, scales, and eyelash shedding [1]. Its manifestations are often similar to bacterial blepharitis, which can easily lead to misdiagnosis. The main reason for the rarity of this disease in clinical practice is the local defense mechanism of the eyelids: antifungal components (e.g., lysozyme) in tears and frequent eyelid movements are not conducive to fungal colonization. Its occurrence often requires the presence of local or systemic susceptibility factors. The key feature of microscopic diagnosis is the discovery of a large number of hyphae and spores enveloping the eyelash hair shaft, which is completely different from other types of fungal infections.

This report focuses on a case of tinea blepharociliaris, emphasizing the rare clinical features of tinea blepharociliaris and the importance of medical history in diagnosis. At the same time, the importance of early differential diagnosis of bacterial blepharitis, tinea blepharociliaris, and other fungal blepharitis was emphasized to ensure the correct treatment and prevent the aggravation of the disease.

## Case presentation

A 25-year-old female patient was referred to our clinic with symptoms that included a six-month-old pruritic erythema of the skin around the eyes and multiple two-week-old scabs on the left upper eyelid following scratching. The patient's medical history revealed prior incidents of cosmetic exposure, occurring within the past six months. Previously, the patient used 0.2 g fusidic acid cream, once in the morning and evening, for five days, and 0.2 g glucocorticoid cream, once a day, for five days, for empirical antibacterial and anti-inflammatory combination therapy. Fusidic acid covered common skin Gram-positive bacteria, and glucocorticoids were used to control the inflammatory response. After treatment, the lesions did not improve, and they showed progressive aggravation. The treatment response was inconsistent with the typical bacterial infection, suggesting that there may be other pathogens (e.g., fungi) infection or that the current treatment is insufficient. The patient denied a history of systemic fever, visual impairment, and immune deficiency.

**Figure 1:**
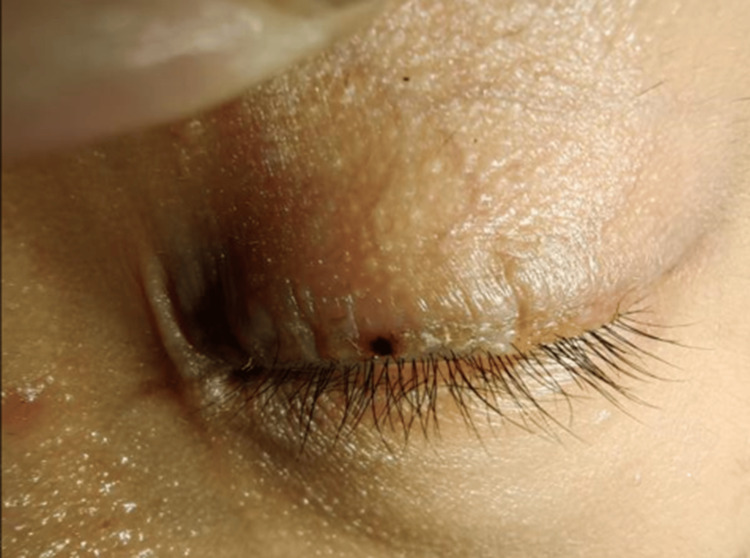
Clinical presentation: mildly scaly erythema of the skin around the eyes and a sesame-sized brown scab on the left upper lid

Physical examination revealed mildly scaly erythema of the skin around the eyes, a sesame-sized brown scab on the left upper lid that was firmly attached and not surrounded by pustules, no broken or detached eyelashes, and dermatoscopy did not show the typical spiral-like changes (Figure 1). Eyelashes are not affected, and the conjunctiva is not congested. The results of the routine laboratory investigations, incorporating analyses of blood and urine, as well as assessments of liver and renal function, and immunological parameters, were found to be within normal limits.

**Figure 2:**
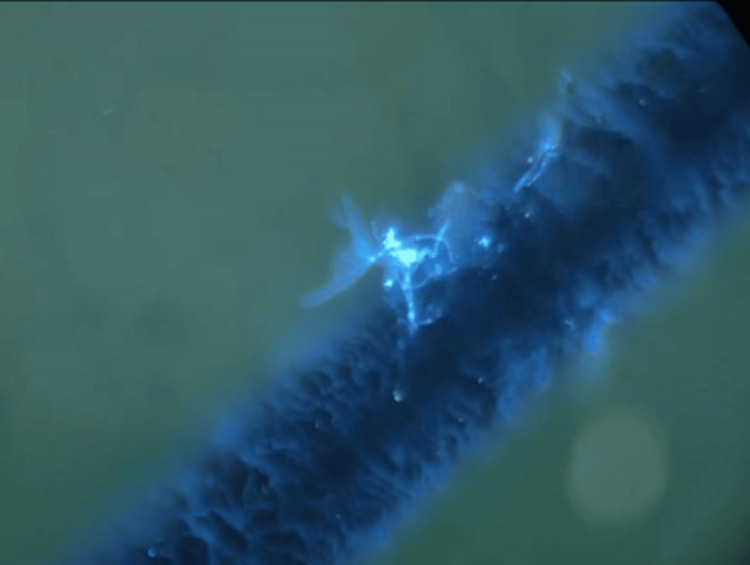
Microscopic examination showing the rod-shaped macroconidia and microconidia spores

In view of the fact that the patient's previous standardized application of anti-bacterial drugs and glucocorticoid therapy proved unsuccessful in alleviating the symptoms, and the lesions exhibited progressive exacerbation, it can be deduced that the initial hypothesis of targeting bacterial infection was unsuccessful. In light of the suboptimal response of the periocular skin to conventional treatments, the distinctive characteristics of the lesion (recalcitrant crusting, the absence of pustules characteristic of bacterial infections), and the relatively warm and humid anatomical environment of the region, the likelihood of infection by atypical pathogens was clinically suspected. A review of the literature identified fungal infection as an important differential diagnosis. In order to clarify the pathogenetic diagnosis, a targeted mycological examination was then performed (including direct microscopy). Microscopic examination revealed macroconidia and microconidia. Multiple rod-shaped macroconidia were scattered, some with septa, appearing uniformly translucent with thin, transparent walls. Most were curved with rounded ends. Microconidia distributed along hyphae were spherical, pear-shaped, or teardrop-shaped (Figure 2). According to the results of microscopic examination, patient history, and clinical manifestations, the final diagnosis was tinea blepharociliaris. The patient was administered oral terbinafine 250 mg/day for two weeks, after which all cosmetic products and hormonal ointments were discontinued. The patient was instructed to cleanse the lid margins with saline only. Complete recovery was observed after the 14-day course, and no recurrence was noted during the two-month follow-up period. No hyperpigmentation or scarring of the affected area was observed.

## Discussion

Blepharitis is a chronic inflammatory disease with complex eyelid symptoms, and the traditional aetiology of blepharitis focuses on bacterial colonisation (mainly staphylococcal infections) and helminthic mite parasitism [8], with the two aetiologies interacting with each other [9]. However, it is worth noting that, in cases where conventional treatment is ineffective, some stubborn cases may be driven by skin ringworm infection. This type of fungal blepharitis has been overlooked for a long time due to two major characteristics: firstly, the eyelids are unusually thin (0.55 mm) compared to other facial areas (2 mm), making them more susceptible to allergen invasion [10]. Fungal infections are rare in typical circular lesions (central regression and raised scales), but often present as non-specific erythema and desquamation. Secondly, misuse of glucocorticoids can mask inflammatory signs, leading to eczema-like or pustular-like appearance of the lesions, further interfering with diagnosis [11].

Dermatophytosis is an infection of the skin, hair, or nails caused by dermatophytes, a group of superficial filamentous fungi that cause fungal infections of the skin. These are also known as ringworms, which mainly parasitise the human stratum corneum, hair, and nail plate. The primary pathogenic dermatophytes include over 40 species such as *Trichophyton*, *Microsporum*, and *Epidermophyton*. They are typically transmitted through direct contact or animal-to-human routes [12]. Trichophyton can infect eyelashes and eyelids, also known as tinea blepharociliaris. Its clinical manifestations are often non-specific blepharitis, such as itching, erythema, scales, and eyelash shedding. Its manifestations are often similar to bacterial blepharitis, which can easily cause misdiagnosis. It is worth noting that diseases such as atopic dermatitis, herpetic eczema, seborrheic dermatitis, psoriasis, and seborrheic pemphigus may also present with similar symptoms such as erythema and scaling. However, these diseases can usually be distinguished from blepharitis by the extent and distribution characteristics of their affected areas. In view of the high incidence rate of blepharitis (whether anterior or posterior) and its typical characteristics of focal involvement, it should be taken as the primary differential diagnosis together with contact dermatitis [13].

If skin ringworm infection around the eyes is detected early, short-term treatment can have a good therapeutic effect on papules, pustules, and periocular desquamation caused by dermatophyte disease. However, fungal infection may be related to various clinical manifestations according to its invasive location, variable invasive ability, personal immunity, diagnosis and treatment procedures, which can easily lead to misdiagnosis and mistreatment, so that the disease cannot be treated in the early stage [14]. In addition, among conventional treatments for bacterial blepharitis, antibiotic formulations have frequently been used to deliver local therapy with limited risk for adverse effects (e.g., bacitracin and erythromycin ointments). Given the infectious and inflammatory nature of blepharitis, topical steroids may help control acute exacerbations. For improving signs and symptoms of blepharoconjunctivitis, combination therapy with steroids proved more effective than azithromycin alone [15]. The use of these medications may further contribute to outbreaks of fungal blepharitis lesions and more severe consequences. This may start a vicious cycle. Firstly, the symptoms may decrease or disappear, since topical corticosteroids mask the response of immunity (erythema, itching, scaling, burning sensation) by suppressing local defence mechanisms. This instead accelerates the growth of fungi. At the same time, because steroids cannot cure the disease, fungal infection intensifies, and soon the disease caused by fungi relapses, patients may use previously helpful drugs or use local corticosteroids again for treatment. Every attack of the symptoms may be more severe than before because, during every treatment with local corticosteroids, which induce local immunosuppression, the growth of the hyphae may extend into the depth [11].

Another specialized study on pityriasis versicolor showed that among 107 patients, 57.1% (61 cases) presented with typical manifestations, while 42.9% (46 cases) exhibited atypical forms. This clinical diversity further complicates diagnosis. It is estimated that approximately 70% of patients with tinea blepharociliaris are misdiagnosed as other skin conditions at initial presentation [16], highlighting its tendency to mimic various inflammatory or infectious lesions. Thus, obtaining an accurate history and completing differential diagnosis in the early stages is crucial to prevent protracted illness or progression to chronicity. Once determined, oral antifungal agents are required for systemic and sufficient treatment time. Eyelid hygiene is still the basis of most treatment programs [15], and patients should be reminded to pay attention to eyelid hygiene habits. At the same time, in order to prevent reinfection, patients should check and treat dermatophytosis of livestock and other body parts.

Direct examination of scales and hair for mycological testing should be conducted whenever available [13]. In addition, it is important to recognize the limitations of direct microscopy. Firstly, this method typically cannot accurately identify fungal species based on morphological characteristics alone. Secondly, the accuracy of results heavily depends on specimen quality, operator experience, and the thoroughness of microscopic examination, potentially leading to false-negative outcomes. When clinical presentation strongly suggests fungal infection but microscopy yields negative results, the diagnosis should not be readily excluded. Further confirmation via fungal culture or molecular biology methods is warranted. The preliminary fungal category identified by microscopy (e.g., distinguishing between yeast hyphae or dermatophytes) may guide empirical treatment, but precise therapy ultimately requires species-specific identification, because antifungal drug sensitivity may vary by species.

Given the unique nature of tinea palpebrae, we recommend heightened vigilance for atypical ocular symptoms. For persistent cases, prompt fungal testing should be initiated to enable early treatment and prevent disease progression.

## Conclusions

This report delineates a case of tinea blepharociliaris, accompanied by the hallmark clinical manifestations of the condition. Tinea blepharociliaris, a rare condition characterized by inflammation of the eyelids, is susceptible to misdiagnosis as eczema or bacterial infection. It is important to note that cases of cosmetic contamination may present as a novel source of infection in urban populations. The key enlightenment of this case is that, if the patients with eyelid inflammation who are ineffective in conventional anti-bacterial therapy and glucocorticoid therapy and whose skin lesions are progressively aggravated, especially for those patients with multiple, needle like (1-2 mm), firmly adherent yellowish brown scabs around the edge of the ciliary body, accompanied by different degrees of desquamation, erythema and eyelash loss, they should be highly alert to the possibility of fungal infection. At this juncture, timely mycological microscopy is paramount for arriving at a definitive diagnosis. As demonstrated in this case, the process of microscopy is both straightforward and expeditious, allowing for direct observation of the characteristic fungal structures (e.g., rod-shaped macroconidia and microconidia). This provided a substantial foundation for the expeditious diagnosis of fungal blepharitis, and the subsequent short-term oral terbinafine treatment achieved a noteworthy level of efficacy in this case.

Although tinea blepharociliaris often presents with symptoms similar to those of common blepharitis, the specific medical history mentioned in this case (e.g., exposure to cosmetics) and atypical skin lesion characteristics (e.g., distinctive sesame-seed-sized brown crusts) provide key diagnostic clues. Particularly when conventional bacterial treatment proves ineffective, the patient's crust characteristics should be regarded as a potential indicator of tinea blepharociliaris. It is therefore recommended that all patients with recalcitrant blepharitis undergo detailed questioning regarding their history of cosmetic and pet exposure. Increased vigilance for fungal infections is also advised, and mycological microscopy should be incorporated into the routine examination process. The heightened awareness of the condition in clinical practice has the potential to prevent the unnecessary use of hormones and the development of chronic conditions.

## References

[1] Bernardes TF, Bonfioli AA (2010). Blepharitis. Semin Ophthalmol.

[2] Turnbull AM, Mayfield MP (2012). Blepharitis. BMJ.

[3] Wang C, Dou X, Li J, Wu J, Cheng Y, An N (2021). Composition and diversity of the ocular surface microbiota in patients with blepharitis in northwestern China. Front Med (Lausanne).

[4] Nguyen B, Hu JK, Tosti A (2023). Eyebrow and eyelash alopecia: a clinical review. Am J Clin Dermatol.

[5] Veldman P, Colby K (2011). Current evidence for topical azithromycin 1% ophthalmic solution in the treatment of blepharitis and blepharitis-associated ocular dryness. Int Ophthalmol Clin.

[6] Brasch J (2010). Pathogenesis of tinea. J Dtsch Dermatol Ges.

[7] Ely JW, Rosenfeld S, Seabury Stone M (2014). Diagnosis and management of tinea infections. Am Fam Physician.

[8] Dougherty JM, McCulley JP (1984). Comparative bacteriology of chronic blepharitis. Br J Ophthalmol.

[9] Zhu M, Cheng C, Yi H, Lin L, Wu K (2018). Quantitative analysis of the bacteria in blepharitis with Demodex infestation. Front Microbiol.

[10] Ferček I, Ozretić P, Tambić-Andrašević A (2024). Comparison of the skin microbiota in the periocular region between patients with inflammatory skin diseases and healthy participants: a preliminary study. Life (Basel).

[11] Sahin GO, Dadaci Z, Ozer TT (2014). Two cases of tinea ciliaris with blepharitis due to Microsporum audouinii and Trichophyton verrucosum and review of the literature. Mycoses.

[12] Rinaldi MG (2000). Dermatophytosis: epidemiological and microbiological update. J Am Acad Dermatol.

[13] Martínez-Ortega JI, Mut Quej JE, Medina Angulo TK (2024). Tinea blepharociliaris: a case report and literature review. Cureus.

[14] Barac A, Stjepanovic M, Krajisnik S, Stevanovic G, Paglietti B, Milosevic B (2024). Dermatophytes: update on clinical epidemiology and treatment. Mycopathologia.

[15] Duncan K, Jeng BH (2015). Medical management of blepharitis. Curr Opin Ophthalmol.

[16] Lin RL, Szepietowski JC, Schwartz RA (2004). Tinea faciei, an often deceptive facial eruption. Int J Dermatol.

